# Enhancing Vibrational Spectroscopy-Based Diagnosis through Bottom-Up Modeling: The Case of Infrared Absorption Spectrum of Urine

**DOI:** 10.1021/acs.analchem.5c06845

**Published:** 2026-04-18

**Authors:** Víctor Navarro-Esteve, Ángel Sánchez-Illana, José Portolés, Maria Marques-Vidas, Antonio J. Sanchez-Lopez, Josep Ventura, Hugh J. Byrne, Bayden R. Wood, David Pérez-Guaita

**Affiliations:** † Department of Analytical Chemistry, 16781 University of Valencia, 46100 Burjassot, Spain; ‡ 16370 University Hospital Puerta de Hierro de Majadahonda, 28222 Majadahonda, Spain; § Department of Medicine, Universidad Autónoma de Madrid, 28029 Madrid, Spain; ∥ Neuroimmunology Unit, Instituto de Investigación Sanitaria Puerta de Hierro-Segovia de Arana, 28222 Majadahonda, Spain; ⊥ Biobank, Instituto de Investigación Sanitaria Puerta de Hierro-Segovia de Arana, 28222 Majadahonda, Spain; # University Hospital Doctor Peset Aleixandre, 46017 Valencia, Spain; ∇ Physical to Life Sciences Research Hub, FOCAS, 8819 Technological University Dublin, Aungier Street, Dublin D02 HW71, Ireland; ○ Monash Biospectroscopy Group, School of Chemistry, Monash University, 3800 Victoria, Australia

## Abstract

Vibrational spectroscopy has become a valuable tool for biomedical applications. However, the technique generates large empirical spectral data sets, requiring multivariate analysis using an ever-evolving arsenal of machine learning approaches. Optimizing and validating these algorithms requires high-quality, appropriately representative data sets with a well-defined diagnostic profile, which are not readily available for testing. This work introduces an *in silico* framework for building and validating spectroscopic diagnostic models using mixture-based spectral simulations, illustrated through infrared urine analysis for diagnosing diabetic kidney disease (DKD). We adopt a bottom-up approach, using a priori biological information related to the disease under investigation to construct a synthetic spectral model of liquid urine, enabling the prediction of diagnostic performance using only literature-derived parameters. Experimental variables, including the protein preconcentration device, measurement time, and instrument settings, were virtually simulated and optimized, generating large and diverse data sets that capture representative ranges of experimental and patient conditions. The resulting data sets were then used to construct machine learning models for predicting albumin and creatinine concentrations. These models obtained with the bottom-up methodology were experimentally validated by comparing with models built with artificial (*N* = 49) and real urine samples (*N* = 61, DKD case–control study), obtaining no significant differences in the analytical figures of merit. The proposed approach eliminates the need for resource-intensive empirical data sets and enables systematic performance prediction and exploration of critical parameters, facilitating rapid, application-specific optimization of vibrational spectroscopy workflows capable of generating over 200 spectra in less than 1 s on a standard desktop computer.

## Graphical abstract

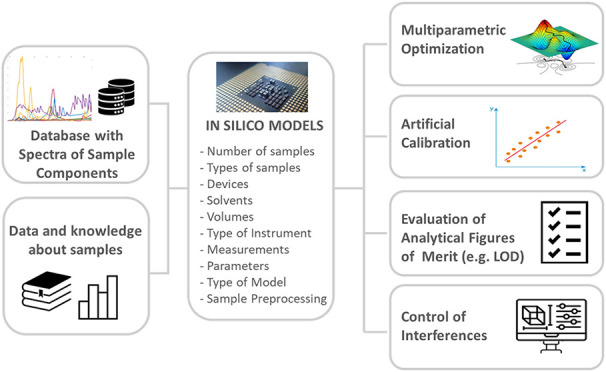

Vibrational spectroscopy is among the most promising techniques in the clinical field.^1–4^ Infrared (IR) and Raman spectroscopy have shown great potential in the quantification of clinical parameters^5^ and the diagnosis of diseases, including malaria,^4^ COVID-19,^6^ and diabetic kidney disease (DKD).^7^ Both techniques are noninvasive methods that offer insights into the molecules participating in biological processes.

However, the development of vibrational methodologies for analyzing complex biological samples remains significantly hindered by the nontrivial task of extracting meaningful information from label-free analyses. The information about the sample composition is generally encrypted in a set of overlapping bands from the constituent biochemical components, which can be perturbed by their local environment or the effect of physiological processes. The use of complex machine learning (ML), although extremely effective in extracting this information,^8^ always requires a prior process by which a representative spectral set containing well-characterized samples is obtained and measured.^9^ This phase is the most laborious in the development of the analytical procedure, requiring the collection of a representative set of clinical samples measured using both vibrational and reference methodologies,^5^ thereby impeding the proper optimization of analytical workflows.

Design of experiments (DOE) strategies provide efficient ways to optimize analytical methodologies.^10^ Furthermore, ANOVA-simultaneous component analysis (ASCA) and related techniques allow assessing the influence of experimental variables on complex spectral responses.^11,12^ However, when working with clinical samples, obtaining data sets that cover a representative population range and various combinations of sample pretreatment steps^13^ or spectroscopic instrumentation^14–16^ factors and levels becomes practically unfeasible. This limitation hinders the development of label-free vibrational methods, which require experimental remeasurement of the whole data set for every change in pretreatment or instrumentation.

In practice, researchers typically focus on a defined analytical problem (e.g., the diagnosis of a particular disorder) and select suitable instrumentation, a simple or null sample pretreatment procedure, and a set of technical measurement parameters (e.g., the number of scans and resolution). Subsequently, significant resources are invested in collecting a calibration set with the hope that the chosen conditions will yield a prediction model with acceptable results. If not, researchers must then try to identify potential pitfalls, address issues related to sensitivity, reproducibility, or the presence of interferences, and measure the calibration set again with the refined method.

In this study, we propose the use of data-driven optimizations, based on experimental spectra and linear combinations, designed to develop and validate models that replicate or predict the empirical behavior of biological systems, as measured using IR absorption spectroscopy. The proposed bottom-up approach builds spectra by combining the measured spectra of individual pure components found in the sample above the limit of detection (LOD). Therefore, the occurrence of a specific disorder can then be simulated by adjusting the concentrations of relevant components according to the literature-reported changes associated with the disease.

This strategy allows the creation of large data sets of synthetic spectra that can be treated with ML techniques in the same manner as experimental data, holding a wide range of potential applications. For example, prediction errors can be estimated by simulating the effects of different analytical parameters on the spectra. Similarly, the methodology itself can be optimized by assessing how variations in these parameters influence the spectral output. This includes factors such as sample interferences, measurement conditions (e.g., the time and spectral resolution), instrumentation type, technical and biological interferences, or the use and type of preconcentration devices. In this way, prediction errors for a given disease under specific experimental conditions can be anticipated, or the *in silico* data set could even be employed as a calibration set.

Multivariate calibration approaches such as partial least squares (PLS) and PLS discriminant analysis (DA) based on single-component reference spectra have been widely reported for the quantitative analysis of complex biological matrices.^17,18^ However, the novelty of this work lies in the integration of multivariate calibration within a comprehensive computational framework for the urine spectrum simulation. This workflow includes the study of instrument-specific response characteristics, noise structure, preconcentration factors, and matrix-related spectral contributions.

To exemplify this concept, we generated multicomponent attenuated total reflectance-Fourier transform IR (ATR-FTIR) spectra of liquid urine and applied the bottom-up framework to develop diagnostic models for DKD. DKD is characterized by albuminuria (the presence of albumin in urine),^19^ which can be measured through protein extraction and preconcentration via ultrafiltration, followed by drying the isolated protein fraction on an ATR crystal for measurement. However, although highly effective for albumin quantification,^20,21^ this approach is unsuitable for screening applications that require rapid spot urine measurements. In such contexts, creatinine quantification is also needed to calculate the urinary albumin-to-creatinine ratio (UACR). However, because creatinine is removed during the protein isolation step,^20^ it is necessary to improve the LOD of the method and reach microalbuminuria levels. Simultaneous quantification of albumin and creatinine requires identifying the optimal pretreatment that balances sensitivity for both analytes.

This task is particularly challenging due to the numerous variables involved, including the optimization of protein preconcentration and the selection of an appropriate spectrophotometer, balancing point-of-need applicability with analytical sensitivity and specificity. In this work, we applied our bottom-up approach to optimize and predict the classification performance when modifications to our ATR-FTIR analytical method in the context of DKD diagnosis were performed. To this end, we defined three types of samples: (i) simulated spectra (generated by combining the individual spectra of urine components), (ii) artificial urine spectra (experimentally obtained by mixing standards of the main components), and (iii) real urine spectra (collected from patients and controls). Experiments were conducted to assess whether the simulated spectra could reliably reproduce experimental data and to evaluate the feasibility of using this approach to optimize the diagnostic performance of the method *in silico*. The widespread adoption of such models would enable researchers and practitioners to gain deeper insights into the systems under investigation and to perform a wide range of experiments without relying on resource-intensive laboratory procedures.^22–26^

## Experimental Section

### Chemicals and Materials

Artificial urine was prepared following the protocol outlined by Worramongkona et al.^27^ CaCl_2_·2H_2_O, KCl, Na_3_C_6_H_5_0_7_·2H_2_O, NaH_2_PO_4_·2H_2_O, NH_4_Cl, glucose, urea, and bovine serum albumin (BSA) were obtained from Thermo Scientific (Waltham, USA). MgSO_4_·7H_2_O and Na_2_HPO_4_ were obtained from Honeywell Fluka (Charlotte, NC, USA). Na_2_SO_4_ and creatinine were acquired from Merck (St. Louis, USA). NaHCO_3_ and NaCl were purchased from Scharlab (Sentmenat, Barcelona, Spain). Ultrapure water (18 MΩ·cm^–1^) was obtained from an Adrona purification system.

A total of 49 artificial urine samples were prepared by combining the 13 components listed above at different concentrations, within the physiological ranges reported in the literature.^28–32^ These multicomponent mixtures followed Brereton’s seven-level design with mutually orthogonal concentrations^33,34^ (see Table 1), allowing independent evaluation of the effect of each component.

**Table 1:** Concentrations of the Components Mixed for the Preparation of 49 Artificial Urine Samples with an Orthogonal Design

Cal components	range according to	reference range (min-max (mg/L))	calibrate range (min-max (mg/L))	refs
CaCl_2_·2H_2_O	Ca^2+^	24.46–611.39	12.23–917.09	^28,29^
creatinine	creatinine	400.67–1957.33	267.11–2348.8	^28^
KCl	K^+^	844.91–3826.97	563.28–4592.36	
MgSO_4_·7H_2_O	Mg^+^	344.71–1818.19	229.81–218.83	
Na_3_C_6_H_5_0_7_·2H_2_O	Na_3_C_6_H_5_O_7_	225.54–1215.47	150.36–1458.56	
NaCl	Na	1597.36–8843.92	1064.91–10612.7	
NaH_2_PO_4_·2H_2_O	PO_4_	2080.08–5200.2	1386.72–6240.24	
Na_2_SO_4_	SO_4_	662.85–4450.59	441.9–5340.7	
urea	Urea	6666–23,333.33	4444–28,000	
NH_4_Cl	NH_4_	535–1997.33	356.67–2396.8	
albumin	albumin	30–3000	0–300	^30^
glucose	glucose	250–20,000	0–750	^31^
NaHCO_3_	HCO_3_	33.6–428.44	28–514.12	^32^

### Real Urine Collection

Spot urine samples were obtained from our previous international multicentric case-control study on the diagnosis of DKD with ATR-FTIR.^21^ In this case, only samples corresponding to the Spanish centers (*N* = 61) were used for simplicity. Those samples correspond to DKD patients (*N* = 35, i.e., the DKD group) and healthy controls (*N* = 26). All samples were obtained with the appropriate approval of the Ethics and Scientific Committees (Ethics Committee of the University of Valencia, code 2425449, and Hospital Puerta de Hierro de Majadahonda Biobank, code CE-0128–2023).

Reference values of albumin and creatinine were obtained via immunoturbidimetry with the Microalbumin Reagent Kit 08P04 and via a kinetic alkaline picrate assay with the Creatinine Reagent Kit 07P99, respectively. Reference values of urea and glucose were obtained via an enzymatic assay with the Urea Nitrogen Reagent Kit 08P16 and the Glucose Reagent Kit 07P55, respectively. Reference values of sodium, potassium, and chlorine were obtained using ion-selective electrodes with the ICT Sample Diluent Kit 07P53. Reference values of phosphorus were obtained via the phosphomolybdate methodology with the Phosphorus Reagent Kit 08P40. An Alinity c autoanalyzer (Abbot, USA) was employed for all of these quantifications.

### Sample Preparation

Protein preconcentration was carried out by transferring 500 μL of urine (artificial or real) into a Vivaspin 500 10 kDa centrifuge filter unit (Sartorius, Göttingen, Germany), prewashed according to the manufacturer’s instructions. Samples were centrifuged at 14,000*g* for 15 min, and the concentrate was collected. For real urine, 700 μL was drawn into a needleless syringe and filtered through a 0.2 μm nylon syringe filter (Scharlab, S.L., Barcelona, Spain) prior to protein preconcentration.

### ATR-FTIR Measurements

Spectra were measured on a PerkinElmer Spectrum Two FTIR spectrometer (Waltham, MA, USA) equipped with a single-reflection UATR accessory. Three μL of the protein concentrate sample was deposited, covering the whole surface of the crystal (no evaporation was observed during the measuring time). The spectrometer was controlled using the Spectrum (v10.7.2) software (PerkinElmer), and the data was corrected using the built-in CO_2_/H_2_O correction. Spectra were recorded from 450 cm ^–1^ to 4000 cm ^–1^ with a resolution of 4 cm ^–1^, averaging the selected number of scans for each condition. A background measurement of the clean diamond crystal was recorded by acquiring the same number of scans.

Artificial urine and standards were also measured on a PerkinElmer Spectrum 3 spectrometer equipped with the DuraSampleIR attenuated total reflection module for liquids with a nine-reflection diamond/ZnSe DuraDisk plate provided by Smiths Detection Inc. (Warrington, U.K.). Due to the bigger size of the surface of the crystal, 6 μL of the wet sample was deposited. The conditions were identical to those used for the Spectrum Two, except that the spectral range was set to 4000–650 cm ^–1^.

### Measurement of Individual Standards of Urine Components and Noise Spectra

Selecting the ideal individual standard concentration for each component is nontrivial. While increasing standard concentrations enhances the signal-to-noise ratio (SNR), it may also introduce nonlinear effects, band shifts, and alterations in band ratios^35^ (Figure S1). Additionally, the solubility of certain compounds in water imposes an upper concentration limit. As a compromise, standard concentrations ranged from 10 to 20 times the physiological urinary levels, and each spectrum was recorded over 50 scans to maximize the SNR (Figure S2). Since the total acquisition time needed was around 5 min, to prevent any possible evaporation that could change the standard concentration, the drop was covered with a glass cap. Additionally, the volume of the drop was increased to 10 μL (for both spectrometers) to minimize the potential concentration changes during the measurement time.

Noise spectra were measured by first acquiring a background spectrum, which was immediately followed by sample acquisition. This procedure was repeated 25 times to calculate the mean and standard deviation of absorbance at each wavenumber, thus obtaining a representative noise spectrum. Measurements were performed using both spectrometers (PerkinElmer Spectrum Two FTIR and PerkinElmer Spectrum 3) with 1, 2, 4, 8, and 10 scans, yielding the corresponding noise spectra for each combination and creating a noise library that was subsequently used in the bottom-up framework. Noise was evaluated using atmospheric spectra rather than aqueous samples, although its magnitude was found to be of the same order (see Figure S3).

### Data Analysis

All data processing and modeling were performed to evaluate the performance and comparability of the simulated, artificial, and real spectral data sets, as well as to assess the feasibility of using *in silico* spectra for optimization and calibration purposes. Spectral generation, data visualization, and analysis were carried out in MATLAB 2024a (MathWorks Inc., Natick, USA) using in-house written scripts and the PLS Toolbox (Eigenvector Research Inc., Manson, USA). All computations were carried out on a standard desktop computer (AMD Ryzen 5600G@3.9 GHz, 8 GB RAM), highlighting the low computational requirements of the proposed approach.

Diagnostic and DKD marker quantification models were carried out using PLS regression and PLSDA, respectively. To compare the performance of the data sets in a reproducible way, the seed for the random number generator was restarted each time. For each model, the optimal number of latent variables (LVs) was selected using 10 split Venetian blinds cross-validation (CV) (i.e., 10-fold CV with systematic interleaving). In all models, weighted least-squares baseline (WLSB), standard normal variate (SNV) normalization, the first derivative, and mean centering were applied to spectra as preprocessing. 1680–1000 cm^–1^ and 1680–1380 cm^–1^ regions were selected for the PLS regression of creatinine and albumin, respectively. For PLSDA (PLS Discriminant Analysis), only the 1770–950 cm^–1^ region was used. The mathematical definitions of the key performance indicators reported throughout the paper can be found in the Supporting Information.

## Results and Discussion

### Spectral Simulation

Spectra were generated following a bottom-up approach (Figure 1), whereby the spectrum of each sample is derived by aggregating the contributions from the spectra of its constituent components, weighted by their respective concentrations. Taking into account the general LOD of the technique, only compounds that present concentrations typically above it are considered. Based on the literature, normal concentration ranges for these components can be used to create concentration data sets. Considering that different diseases or conditions present distinct concentration ranges for some specific metabolites, data sets representing different populations can be constructed.

**Figure 1:**
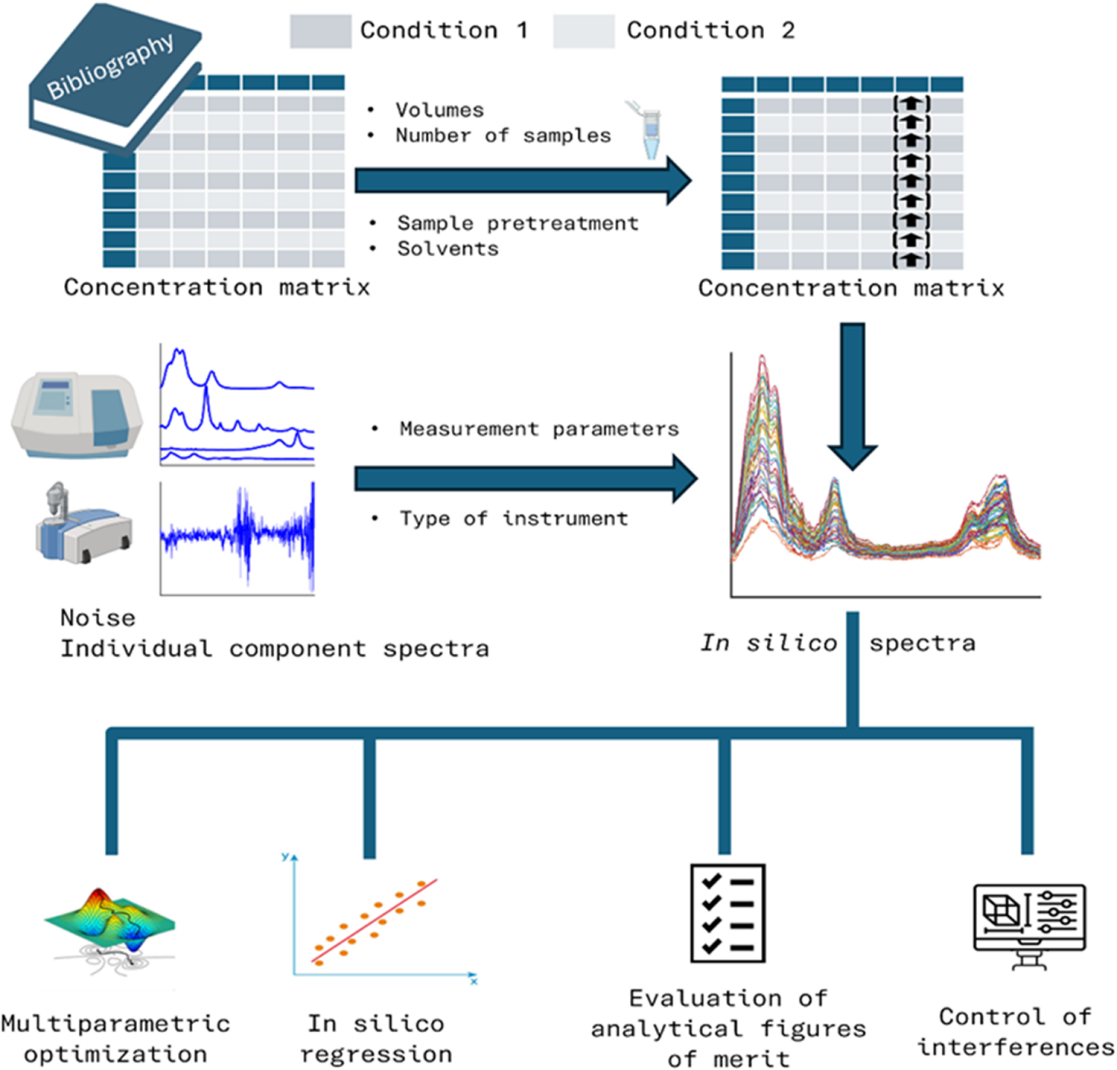
Overview of the bottom-up approach and its potential applications for diagnosis and experimental design optimization.

The bottom-up approach enables the modeling and study of the effect of sample pretreatment steps. The goal of most of them is to preconcentrate the target analytes and/or to decrease the concentration of interferent metabolites.^7,9,13,20,21^ With prior knowledge of the preconcentration/dilution factor, concentrations can be modified accordingly by multiplying by these factors. As the pretreatment methodologies usually present an associated error, this can be considered to calculate a distribution of preconcentration factors *c*(μ,σ). In parallel, pure component spectra were measured at high concentrations to maximize the SNR. These can be measured using several instruments if we want to compare the performance of different spectrometers.

Once final concentrations after sample pretreatment are defined and pure component spectra are measured, the sample spectrum is calculated using eq 1.

1 $$A_{j}=\sum_{i=1}^{n}\left(\frac{c_{i}}{s_{i}}b_{i,j}+\left(1-\frac{c_{i}}{s_{i}}\right)w_{j}\right)+N\left(0,\sigma_{j}\right)$$

where *A*_*j*_ is the absorbance at wavenumber *j*, which is obtained as a linear combination of the absorbances of *n* pure components dissolved in water (*b*_*i,j*_), each scaled by the ratio between the final concentration of the respective constituent (*c*_*i*_) and the concentration at which the standard was experimentally measured (*s*_*i*_). Since all standards were dissolved in water, the water spectrum (*w*_*j*_) is also included in each iteration to account for its contribution, applying the factor $\left(1-\frac{c_{i}}{s_{i}}\right)$ to add or subtract the spectrum of water (*w*_*j*_). White noise (*N*_*j*_) is added following the experimentally defined distribution such that the mean is 0 and the standard deviation is dependent on the number of scans and the spectrometer type, obtained as described in the Experimental Section.

In total, 13 artificial urine components (see the Chemicals and Materials subsection) were used for the bottom-up framework in this study, including CaCl_2_, KCl, and NaCl, inorganic salts that do not absorb in the infrared but can influence the shape and intensity of the water absorption bands.^36^ The MATLAB implementation on a standard laptop generates >200 spectra in less than 1 s.

### Simulated, Artificial, and Real Urine Spectra Overview

To establish the validity of our spectral generation approach, a visual comparison of the artificially created spectra with matching real spectra was conducted, after water subtraction, given that urine is approximately 95% water.^37^ Three data sets were considered. The first data set, constructed through the bottom-up framework, consisted of 49 urine samples following a seven-level design with mutually orthogonal concentrations as described in the Experimental Section and Table 1. The second data set contained 49 spectra from lab-prepared artificial urine, each corresponding to an *in silico* sample and following the same seven-level orthogonal design. The third data set corresponds to the *N* = 26 control group real urine samples.

Thus, each simulated sample has its corresponding twin artificial sample containing the same concentrations of the same constituents. In contrast, the concentration of real samples cannot be controlled, so only the control samples are used in this section for qualitative spectral comparison. Figure 2A depicts the mean spectra of simulated, artificial, and real urine in the fingerprint region. In all cases, the fingerprint region is dominated by bands from urea, creatinine, phosphates, and sulfates (Figure S2C), with prominent peaks at 1626, 1599, and 1466 cm^–1^ for urea, 1100 cm^–1^ for sulfates, and 1080 cm^–1^ for phosphates. The simulated spectra closely matched the artificial urine spectra, confirming the bottom-up approach’s ability to replicate the experimental data. However, small differences became apparent when each experimental spectrum was subtracted from that of its simulated counterpart. Figure 2D shows the results of these subtractions, comparing each measured artificial urine spectrum to its corresponding bottom-up framework using the same component concentrations. The main differences arise from divergences in the hydration level of the samples and nonlinear effects such as water-urea interactions, influencing the 3600–3000 and 900–450 cm^–1^ regions, or minor protein adsorption to the ATR crystal.^38^ Nevertheless, spectral discrepancies are minimal in the fingerprint region, being only a small fraction of the water-subtracted spectra (Figure S4). The mean correlation coefficient between the simulated and artificial spectra is 0.955 in the fingerprint region (1700–1000 cm^–1^). Quantitatively, the RMSE represents, on average, the 9.7% of the subtracted spectra. This value is in part oversized due to the contribution of sections of the fingerprint region with low signal and not so valuable information.

**Figure 2:**
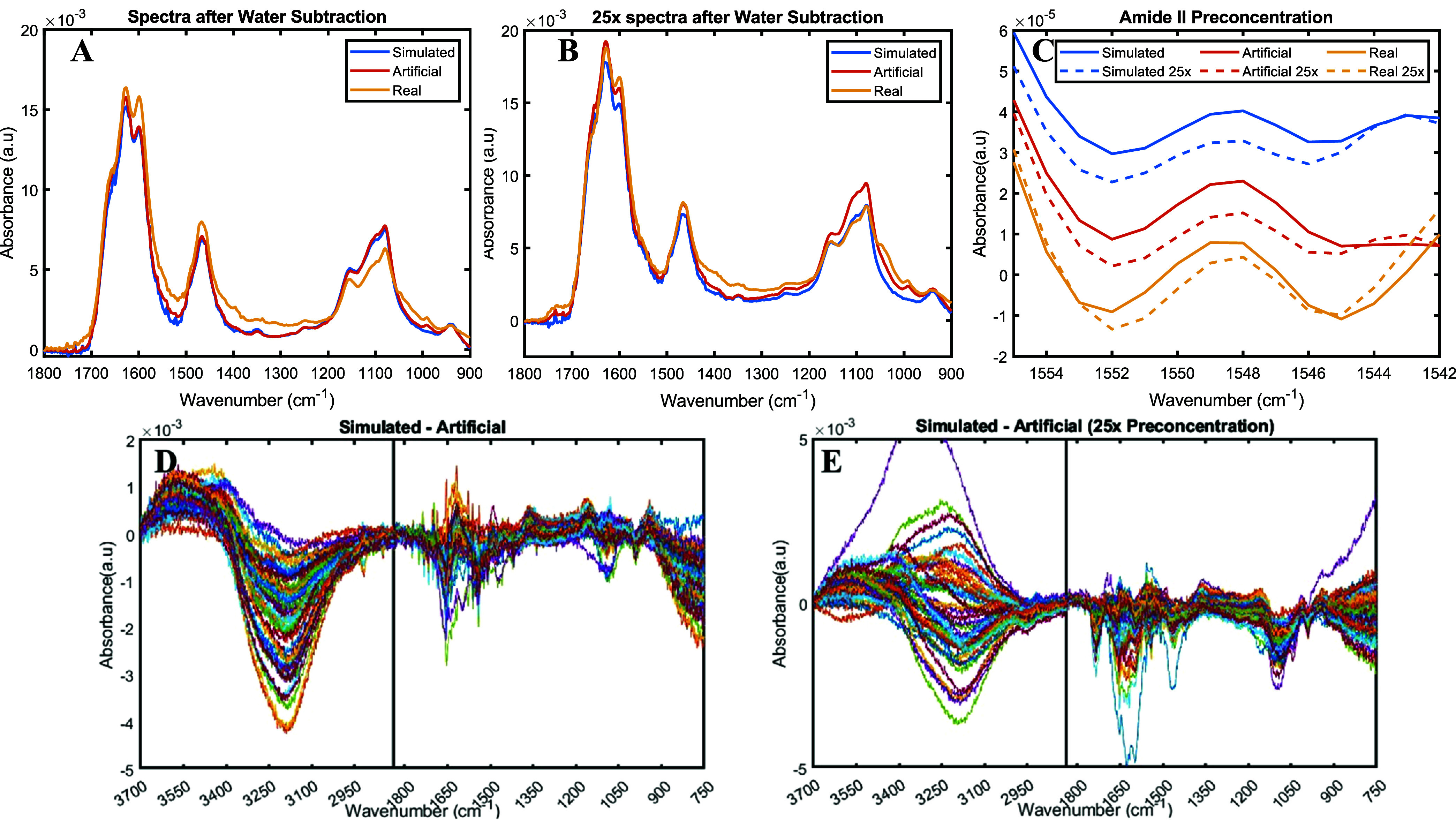
Water-subtracted spectra of real, artificial, and simulated urine before (A) and after (B) preconcentration using 10 kDa filters and their Amide II second derivative offset spectra (C). Subtraction of each measured artificial urine from its corresponding simulated spectra before (D) or after (E) preconcentration.

Strong similarities were also observed when comparing preconcentrated spectra obtained by using the ultrafiltration method (Figure 2B). The effect of preconcentration is highlighted in Figure 2C, which shows a close-up of the 1560–1540 cm^–1^ region. An increase in the Amide II band intensity is evident after concentration, as seen in the average spectra of the three data sets considered. However, subtraction of each preconcentrated artificial spectrum from its simulated counterpart revealed larger deviations (Figure 2E). The occurrence of some Amide bands may be explained by differences in protein content between the simulated and artificial urine samples, as the preconcentration factor of the ultrafiltration devices shows considerable experimental variability (15% RSD). Moreover, membrane contaminants of the preconcentration filters also contribute to the artificial urine spectra. Glycerol, the main contaminant in the ultracentrifuge membrane, is in part responsible for the negative bands found in the 1200–1000 cm^–1^ region, although phosphates may have been affected by preconcentration and influence this region too. Another unidentified contaminant influences the negative carbonyl band at 1735 cm^–1^ (both spectra can be visualized in Figure S5). Moreover, the membrane contaminant has its own influence on the 3600–3000 and 900–450 cm^–1^ regions as it adheres to the diamond and reduces the contact surface between the urine and the ATR crystal, therefore reducing the water bands differences in comparison with the spectra of the pristine samples.

### Design Optimization

Once we demonstrated the comparability between the bottom-up framework and the measured data, we applied this simulation strategy to optimize the determination of the UACR. Sample processing in prior studies involved albumin preconcentration using 10 kDa ultracentrifuge filters and three washing steps to completely isolate the protein fraction of urine.^7,20,21^ In this work, we simulated additional preconcentration and washing steps, which could be carried out with different preconcentration filters with different sample capacity (positively correlated with their price) and, consequently, different preconcentration factors and required sample volumes. The measurement also involved the use of two spectrometers, a compact (Spectrum Two) and a benchtop (Spectrum 3) ATR-FTIR, with acquisition times varying with the number of scans.

*In silico* data sets were generated following the same initial seven-level orthogonal design described before (see Table 1) with concentrations of 0–300 mg/L for albumin (spanning the microalbuminuria range) and 267–2349 mg/L for creatinine (based on literature).^28^ To these base concentrations, different albumin preconcentration factors (1×, 25×, 50×, 100×) and low-weight urine metabolite dilution factors (1×, 0.05×, 0.0025×, corresponding to 0, 1, and 2 cleaning steps, respectively) were applied. Additionally, the preconcentration factor was simulated with a coefficient of variation of 15%, reflecting irreproducibility in the final retentate volume of the ultracentrifuge filter, as indicated by the manufacturer. This irreproducibility value was empirically obtained by weighing 49 ultracentrifuge filters before and after centrifugation. The different numbers of scans (1, 2, 4, 8, and 10) were simulated employing the corresponding experimental noise spectra. The seed of the number random generator was reset for each combination of parameters, so that the noise sampling and albumin preconcentration factor error were identical across the different conditions.

The aim of the optimization was to improve the albumin quantification while retaining sufficient information on creatinine, as both are essential for the determination of the UACR. The results of the RMSECV (root-mean-square error of cross-validation) for the Spectrum Two spectrometer are depicted in Figure 3A,B. This metric was used to assess model performance because it provides an unbiased estimate of prediction error under cross-validation, accounts for model complexity, and is particularly well suited for PLS optimization with limited and heterogeneous data sets. Increasing the number of cleaning steps (i.e., after preconcentration, adding water to the filter and centrifuging again to dilute nonprotein metabolites) did not lead to a statistically significant reduction in albumin RMSECV. This suggests that the PLS does not need the elimination of interferent metabolites to maximize the albumin covariance. On the other hand, even a single cleaning step pronouncedly increased the RMSECV of creatinine by more than a 10× factor. Thus, cleaning steps were categorized as counterproductive for the methodology, even without considering the extra 15 min of sample preprocessing time that each cleaning step requires.

**Figure 3:**
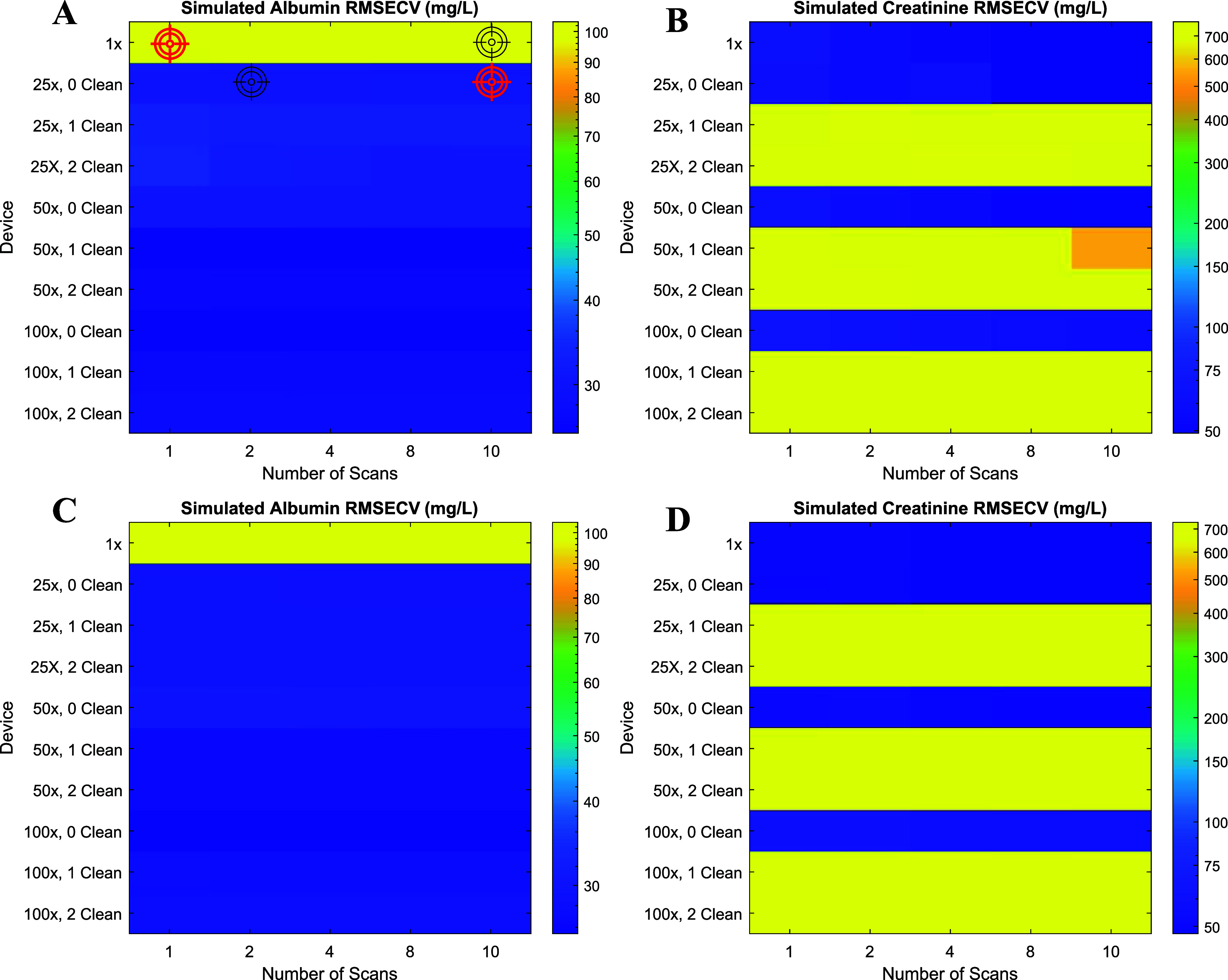
RMSECV of albumin (A, C) and creatinine (B, D) for different numbers of scans in the right axis and different albumin preconcentration factors and cleaning steps for low molecular weight metabolites dilution in the left axis. RMSECV is colored using a logarithmic scale. Conditions selected for comparison with artificial urine are marked with a target icon (A). Measurements were performed on a single-reflection ATR Spectrum Two and in the nine-reflection ATR Spectrum 3.

Regarding the preconcentration of albumin, raw urine (i.e., a preconcentration factor of 1×) showed no meaningful predictive capability and was therefore excluded from further consideration. Increasing the albumin preconcentration factor from 25× to 50× did not yield a significant improvement in albumin quantification performance, while further increasing it to 100× only led to a 14% reduction in RMSECV. However, this may not apply to data sets containing normoalbuminuric samples (<30 mg/L), for which higher preconcentration may be more beneficial. Concerning creatinine, its quantification almost remained largely unaffected by protein preconcentration, increasing the RMSECV only to a 4% and a 17% (when changing from 25× to 50× and 100×, respectively). Therefore, an albumin preconcentration factor of 25× was selected as the optimal compromise between analytical performance and practical constraints, as it requires only 0.5 mL of urine and enables the use of smaller ultrafiltration devices with improved centrifuge throughput. The number of scans had only a minor effect on creatinine prediction (18% decrease in RMSECV from 1 to 10 scans), and a negligible influence on albumin quantification. A setting of 10 scans (1 min of measurement) was selected for subsequent measurements.

To assess whether upgrading to a benchtop spectrometer would be advantageous, we repeated the same measurements and optimization on a Spectrum 3 spectrometer equipped with a nine-reflection ATR device. The corresponding RMSECV values obtained are colored in Figure 3C,D. No significant improvement in albumin or creatinine prediction was observed under any of the tested conditions (<5% decrease in RMSECV). Thus, the increased number of ATR reflections did not translate into improved analytical performance. Given the reduced portability, higher cost, and lack of measurable benefit, the benchtop configuration was deemed unsuitable for point-of-care (PoC) implementation. Consequently, all subsequent experimental work was conducted using the portable single-reflection ATR device, the Spectrum Two spectrometer.

### Comparison with Artificial Urine

To validate the proposed data-driven optimization, artificial urine samples with the same metabolite profile as those of the samples generated through the bottom-up approach were used to contrast the results. As measuring the same samples in 50 different conditions would be extremely time-consuming, we decided to assess the robustness of the bottom-up framework by measuring the artificial urine data set under the four conditions marked with a target icon in Figure 3A: unprocessed urine with 1 scan, unprocessed urine with 10 scans, 25× preconcentration with 2 scans without low-weight metabolites dilution and 25× preconcentration with 10 scans without low-weight metabolites dilution. From now on, these conditions will be referred to as 1 × 1Sc, 1 × 10Sc, 25 × 2Sc, and 25 × 10Sc, respectively. Then, different PLS regression models were constructed using both *in silico* and artificial data sets.

Creatinine quantification proved to be robust across all four selected measurement conditions. Under the 25 × 10Sc setting, calibration curves derived from the simulated and artificial data sets were nearly identical (Figure 4A,B). However, for 1 × 1Sc conditions, some differences began to emerge. Since creatinine concentration remained constant between 25 × 10Sc and 1 × 1Sc, the observed discrepancies are attributable to increased noise levels. Although artificial calibration precision decreased slightly, the data set generated through the bottom-up approach was barely affected by this noise factor (Figure 4E,F). This is clearly illustrated in the regression vector for 1 × 1Sc conditions in Figure S6, showing a slightly noisier profile with a significant number of spikes in the simulated regression vector compared to the artificial one.

**Figure 4:**
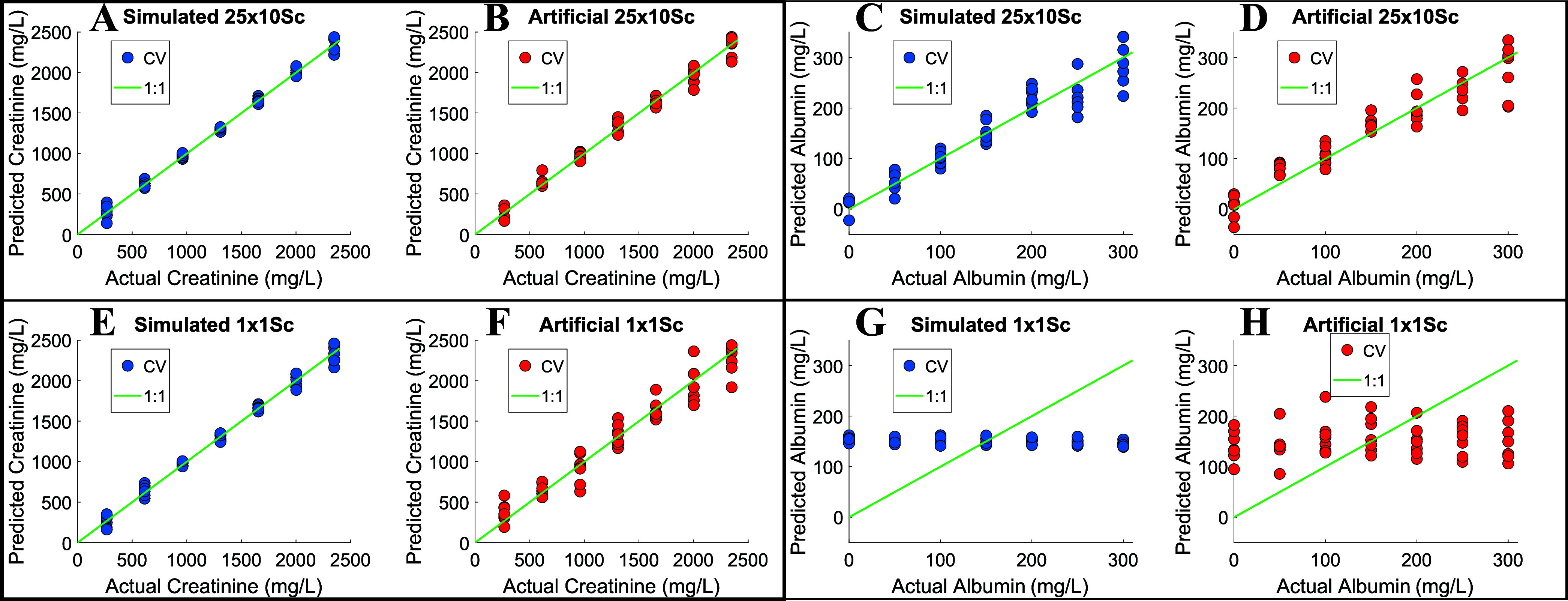
Actual vs CV predicted concentrations of creatinine (A, B, E, F) and albumin (C, D, G, H) for PLS models using simulated (blue dots) and artificial (red dots) data sets under experimental conditions 25 × 10Sc and 1 × 1Sc. These correspond to the red target icons in Figure 2A.

RMSECV and *R* ^2^ for the artificial and simulated PLS models can be seen in Table S1. Overall, *in silico* models slightly outperformed their artificial counterparts, but both exhibited similar trends. The 25 × 10Sc and 1 × 10Sc conditions yielded comparable performance, whereas RMSECV increased for 25 × 2Sc and 1 × 1Sc. Thus, this confirms the number of scans as the most important parameter for creatinine quantification independently of protein preconcentration. When the 25 × 10Sc integrated regression vectors, variable importance in projection (VIP) scores, and integrated first orthogonal loading are compared in Figure 5A–C, these are almost identical, preserving the same band positions in both simulated and artificial models. Minor differences arise from baseline shifts in some bands.

**Figure 5:**
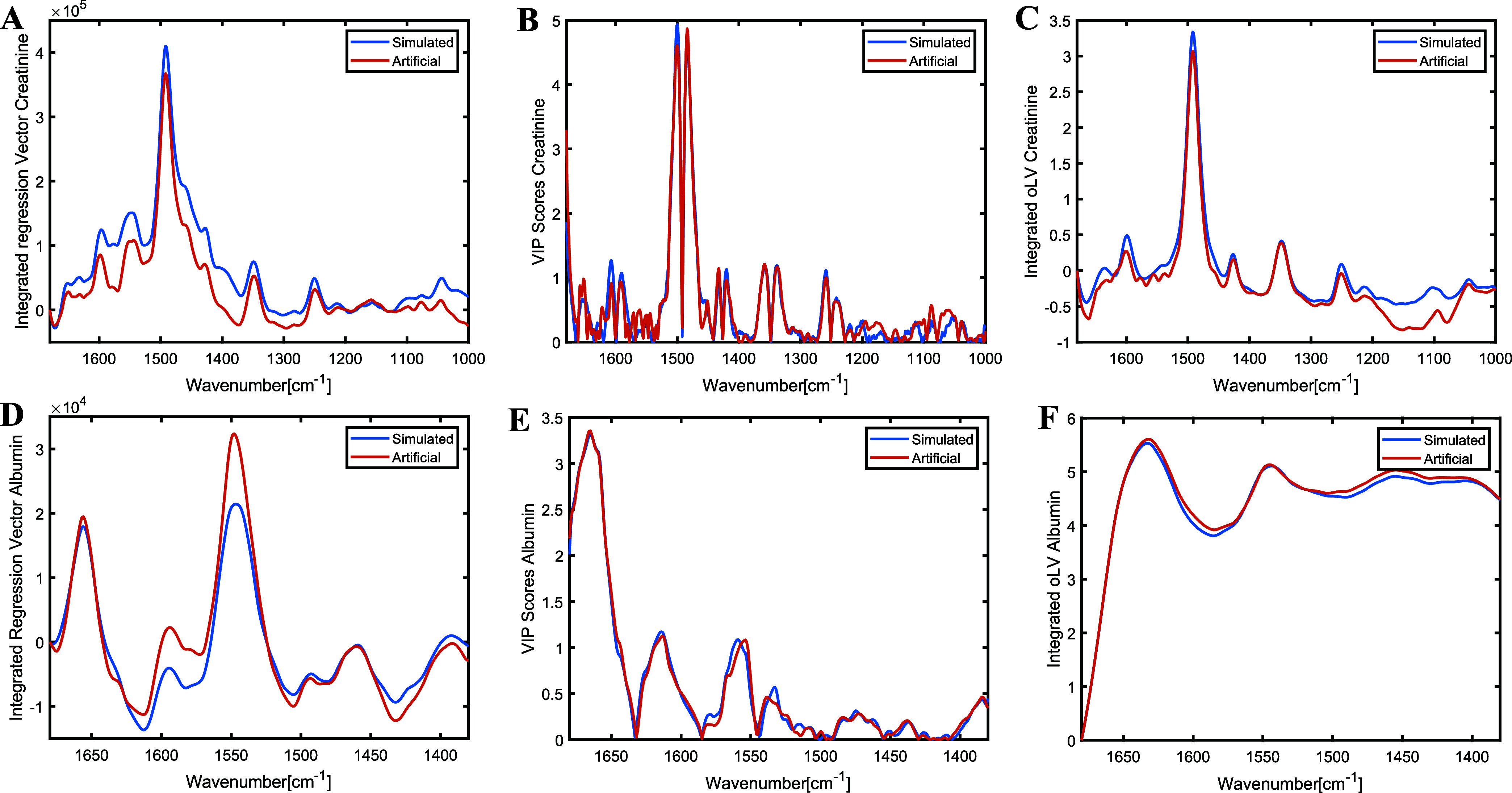
Integrated regression vector, VIP scores, and integrated first orthogonalized loading of the PLS model 25 × 10Sc for creatinine (A–C) and albumin (D–F) calibrated using artificial (blue) and simulated (red) data sets.

Albumin quantification optimization was the most important variable for the success of the methodology, as albumin presents diagnostic capabilities by itself, even without normalizing for creatinine.^21^ Under 25 × 10Sc conditions, satisfactory calibration curves were obtained for both simulated and artificial data sets (Figure 4C,D). Due to the random error in the albumin preconcentration factor, their concentrations are not identical, so the simulated vs artificial comparison is not completely straightforward. Under condition 1 × 1Sc, neither the simulated nor the artificial models were capable of quantifying albumin (Figure 4G,H), as it would be expected due to concentrations below the LOD. This outcome reinforces the credibility of the *in silico* urine model, as it suggests that overfitting to noise is limited under low-signal conditions, thereby increasing the model’s trustworthiness.

The performance of both 25 × 10Sc and 25 × 2Sc models was almost identical for simulated and artificial data sets (Table S1), indicating that the number of scans has a limited impact on albumin quantification. As shown in Figure 5D–F, the regression vector, VIP scores, and the first orthogonal loading pattern were highly consistent between the simulated and artificial models. Again, the band positions aligned well across both models, with only minor differences attributable to baseline shifts in certain bands.

The strong similarity between the bottom-up and artificial loadings is particularly significant, as it demonstrates that the comparable quantitative performance of both models arises from the use of the same molecule-specific wavenumbers in the PLS model construction

### Comparison with Real Urine and *In Silico* Calibration

As 25 × 10Sc was identified as the optimal condition, we processed and measured the real urine data set accordingly. For this purpose, we used a second data set generated through the bottom-up approach, in which we developed 61 synthetic samples aiming to mimic the 61 samples of our real urine data set described in the Experimental Section. For each *in silico* sample, values for urea, creatinine, glucose, albumin, sodium, potassium, phosphorus, and chlorine were obtained from their real sample counterparts, which were measured using standard methodologies at the hospital,^21^ the rest of the components were obtained from normal distributions based on Table 1. This approach, although not perfectly replicating the spectra as in the previous section (due to the unknown concentrations of certain compounds), enabled a reasonable comparison of the RMSEP of both *in silico* and real models.

Figure 6A,B displays the distribution of the PLS RMSEP (RMSE of Prediction) for creatinine and albumin for the *in silico* and real models. A Monte Carlo double CV (MC2CV) was performed by randomly splitting into training and test sets 100 times as in ref 6. The seed of the number random generator was reset so that the splits were the same for both simulated and real data sets. The training test was used to develop the model, and the optimal number of LVs of the model was determined automatically by incrementally adding LVs, provided that the inclusion of the next LV reduced the RMSECV by more than 10%, with an exception if the number of LVs was lower than 5. Then the optimal model was used to predict the independent test set for each split, obtaining a total of 100 different values of RMSEP for creatinine and albumin. The resulting RMSEP distributions shown in Figure 6 exhibited similar means and standard deviations when comparing both models. For creatinine, 278 ± 80 and 285 ± 49 mg/L in the *in silico* and real models, respectively. For albumin, 164 ± 61 and 166 ± 60 mg/L in the *in silico* and real models, respectively. A *t* test was conducted to support that there is not enough evidence to assume that in silico and real distributions are different (*p* = 0.32 and *p* = 0.71 for creatinine and albumin, respectively).

**Figure 6:**
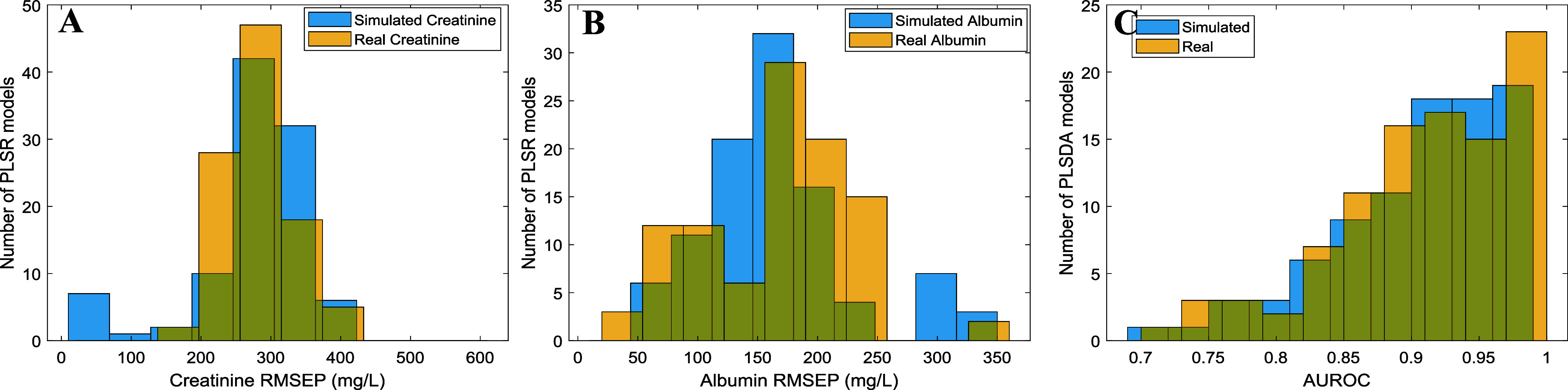
Histograms of RMSEP for the MC2CV PLS models of creatinine (A) and albumin (B). Histogram of the AUROC for the MC2CV PLSDA models (C).

Finally, we evaluated the classification performance of the data sets generated through the bottom-up approach. Similar to the approach employed for regression, two PLSDA were calibrated and validated using *in silico* and real split data sets, aiming to discriminate normoalbuminuric (UACR < 30 mg_albumin_/g_creatinine_) from albuminuric samples. Again, similar distributions for the AUROC (area under the receiver operating curve) were obtained as depicted in Figure 6C, with means and standard deviations of 0.92 ± 0.07 and 0.91 ± 0.08 for *in silico* and real models, respectively. A Wilcoxon rank-sum test was conducted to support that there is not enough evidence to assume that *in silico* and real distributions are different (*p* = 0.49).

Due to the close similarities between the synthetic and real data, our next aim was to explore the potential of *in silico* calibration, that is, whether ML models trained on synthetic spectra can accurately predict the properties of real urine samples. To avoid the use of information about the real data, a new, entirely *in silico* calibration data set was generated using typical component concentrations found in the literature.^38^ It consisted of 100 samples with normal distributions of concentrations for 13 of its components, which better approximate the variability observed in real urine. Among these, urea, creatinine, sulfate, and phosphate were simulated with correlations accounted for (Table S2).^38^ This data set was then used to calibrate a PLSDA model, which was subsequently applied to the real urine data set. Remarkably, with the *in silico* calibration, an AUROC of 0.992 with a prediction classification error of 4.8% was achieved (Figure 7).

**Figure 7:**
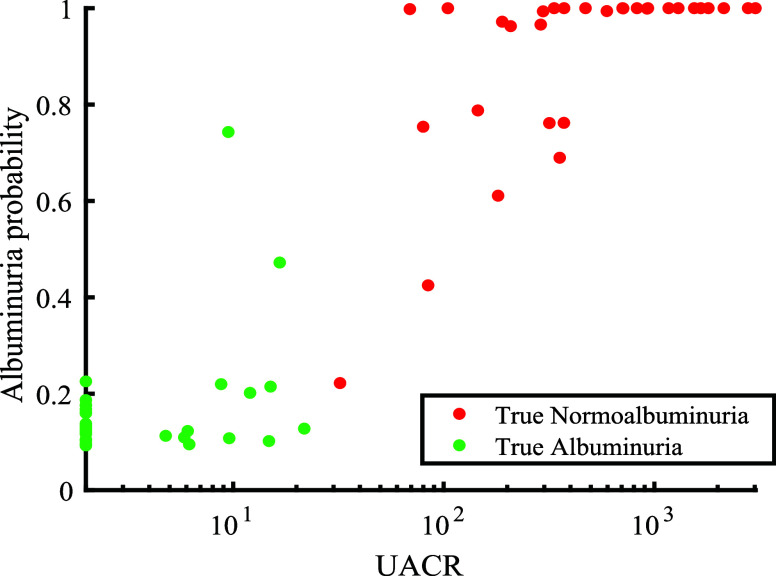
Probability of the real urine samples being categorized with albuminuria represented vs UACR on a logarithmic scale for the *in silico* calibration PLSDA.

### Potential Applications and Limitations

The spectra generated through this bottom-up approach presented only minor discrepancies from those of measured artificial urine. Classification and regression figures of merit were comparable to those from real urine models, demonstrating that these spectral differences are not very relevant to the construction of the chemometric model. Moreover, the analysis of the loadings demonstrated that the similarity between models extends beyond predictive performance, as the same variables were identified as being the most relevant for model construction. IR spectra generated through the bottom-up approach proved to be a valuable tool for analytical design optimization, enabling the systematic evaluation of experimental parameters while minimizing the time, costs, and sample consumption. This strategy allowed us to discard sample pretreatment approaches such as dilution of nonprotein metabolites and to investigate the influence of the albumin preconcentration factor, the SNR, or the spectrometer type. To the best of our knowledge, single-component reference spectra have not previously been employed for this type of multiparametric optimization in complex biofluid analysis. Furthermore, as far as we are aware, this study represents the first methodology proposed for the determination of the UACR using liquid urine measured directly by ATR-FTIR spectroscopy.

However, this strategy presents some notable limitations, such as its dependence on available bibliographic information. In our case study, we knew beforehand that there is a standardized diagnostic criterion for the DKD (the UACR) and the typical concentrations of the most abundant urine metabolites are well documented. Consequently, we could identify albumin and creatinine as the analytes of interest, optimize their spectral signals, and make use of the existing literature detailing their concentrations in both control and DKD samples.

Nonlinear effects or artifacts are frequently encountered in real measurements^39^ but are not accounted for in this approach. Intermolecular interactions, solvation effects, and potential clustering among components are neglected by the linear mixing assumption, which implies some limitations that have to be considered on a case-by-case basis. For the DKD urine case study presented here, the average discrepancy between generated and experimentally measured spectra was 9.3%, which can be largely attributed to such nonlinear effects. These deviations may lead to a reduced prediction performance. Particularly, protein quantification may be affected by its adsorption to the ATR crystal^38^ and by imperfect correction for water vapor contributions.

The framework we follow in this study is, therefore, best suited for the optimization of methodologies with direct measurement of liquid samples (without drying). This way, nonlinear effects are minimized. In contrast, extending this bottom-up approach to dry film measurements would be substantially more challenging, as the drying of urine droplets is a highly complex process^40^ that cannot be adequately modeled by linear combinations of single component spectra. Moreover, although the present strategy proved effective for urine, its direct application to more complex biological matrices, such as blood, is limited.

One advantage of this bottom-up approach is that we can overcome the limitation of the representativeness of the real samples. We can adjust the concentrations of our samples according to the requirements of our optimization design. For example, in this study, we generated samples with mutually orthogonal concentrations, allowing regression models to isolate and quantify the signal specific to each target analyte. This also paves the way for *in silico* calibration, as it enables the generation of personalized data sets with concentration ranges tailored to specific analytical problems. These data sets can then be used as calibration sets to train machine learning models, which in turn can be applied to predict real urine samples.

Furthermore, if the nature of the samples undergoes slight changes (e.g., the disease under study has different subtypes not initially considered),^41^ or some potential interferences emerge, researchers must expand the calibration set accordingly. This bottom-up approach has the potential to be useful in these scenarios, as the number of potential interferents and scenarios in biological samples is substantial, and it is challenging for researchers to assess which would contribute significantly to skewing the model.

In this study, the utility of our proposed bottom-up approach was demonstrated with IR spectroscopy, particularly with the spectra of urine and the example of DKD. However, the same principle should apply to Raman spectroscopy,^23^ although the resulting models would differ substantially and would require validation.

Regarding the analytical performance of the optimized analytical method for DKD monitoring, it should be noted that, although the long-term goal of our methodology is to provide an alternative to urine dipsticks for DKD screening, the reported albumin and creatinine relative errors for the real urine data set are substantially larger than those achieved by the established immunoturbidimetric gold standard methods routinely used in hospital clinical laboratories. These discrepancies can be partly attributed to a limitation for which our bottom-up approach can be more useful during the development of the analytical methodology: the limited size and representativeness of the available real urine data set. The samples analyzed in this study exhibited a very broad range of UACR values, spanning from normoalbuminuria to macroalbuminuria, and the data set was partitioned entirely at random without accounting for sample representativeness. While the MC2CV was useful to demonstrate that the *in silico* and real data sets yield comparable RMSEP distributions, it also resulted in a relatively widespread range of RMSEP values depending on the calibration/validation split. Increasing the size of the real urine data set would be expected to improve model robustness and reduce quantitative errors, potentially approaching the performance observed for the orthogonal design data set. Despite the limitations at this early stage of methodology development, DKD classification performance remains promising. An AUROC greater than 0.9 was achieved using MC2CV, reaching 0.992 for the prediction of the real urine data set when the model was calibrated using the computationally generated data set.

## Conclusions

In this study, we propose a bottom-up approach to generate vibrational spectra. In order to validate this strategy, we generated different *in silico* infrared urine data sets, aiming to use them to guide us through the experimental design optimization of the diagnosis of DKD based on the UACR. The resulting spectra were largely comparable to the artificial urines except for some minor nonlinear effects and additional discrepancies corresponding to membrane contaminants and different preconcentration factors. Sample pretreatment was optimized using this simulated data set and confirmed with experimentally measured artificial urine. Optimal conditions were selected for the processing and measuring of the real urine, obtaining similar quantification and classification errors when compared with *in silico* models. Furthermore, *in silico* calibration allowed the prediction of albuminuria in the real samples. The approach has proven to be a valuable tool in the early stages of experimental design when few or no real samples are available.

## Supplementary Material



## Data Availability

Data sets and details of the data processing can be found at the Zenodo repository (10.5281/zenodo.15798612)

## Notes

Mathematical definitions, concentration ranges, bands shift in highly concentrated solutions, individual components spectra, evaporation effect, noise spectra, contaminants spectra, analytical figures of merit of the PLSR, regression vector comparison, and correlation matrix (PDF)

## Abbreviations

IR: infrared
DKD: diabetic kidney disease
ML: machine learning
LOD: limit of detection
PLS: partial least-squares
PLSDA: partial least-squares discriminant analysis
ATR-FTIR: attenuated total reflectance-Fourier transform infrared spectroscopy
UACR: urinary albumin-to-creatinine ratio
SNR: signal-to-noise ratio
CV: cross-validation
LV: latent variable
RMSE: root-mean-square error
VIP: variable importance in projection
MC2CV: Monte Carlo double cross validation
AUROC: area under the receiver operating curve
