# Supplementary material for: Enhancing Vibrational Spectroscopy-Based Diagnosis through Bottom-Up Modeling: The Case of Infrared Absorption Spectrum of Urine
Source: Anal Chem. 2026 Apr 18;98(17):12328–39. doi: 10.1021/acs.analchem.5c06845 (PMC13150815; doi:10.1021/acs.analchem.5c06845)
Supplement: Supplementary file 1 [file ac5c06845_si_001.pdf]

Supporting Information

Enhancing vibrational spectroscopy-based diagnosis through bottom-up modelling: The case of infrared absorption spectrum of urine

Víctor Navarro-Esteve[1], Ángel Sánchez-Illana[1], José Portolés[2,3], Maria Marques-Vidas[2], Antonio J. Sanchez-Lopez[4,5], Josep Ventura[6], Hugh Byrne[7], Bayden R. Wood[8], David Pérez-Guaita[1]*

[1] Department of Analytical Chemistry, University of Valencia, 46100 Burjassot, Spain
[2] University Hospital Puerta de Hierro de Majadahonda, 28222 Majadahonda, Spain. RISCORS2040 RD24/0004/0028
[3] Department of Medicine. Universidad Autónoma de Madrid, 28029 Madrid, Spain
[4] Neuroimmunology Unit, Instituto de Investigación Sanitaria Puerta de Hierro-Segovia de Arana, 28222 Majadahonda, Spain.
[5] Biobank, Instituto de Investigación Sanitaria Puerta de Hierro-Segovia de Arana, 28222 Majadahonda, Spain.
[6] University Hospital Doctor Peset Aleixandre, 46017 Valencia, Spain
[7] Physical to Life Sciences Research Hub, FOCAS, Technological University Dublin, Aungier Street, Dublin D02 HW71, Ireland.
[8] Monash Biospectroscopy Group, School of Chemistry, Monash University, 3800 Victoria, Australia

*david.perez-guaita@uv.es

Contents

Mathematical definitions S3
Spectral shifts at high concentrations S4
Individual spectra of the urine components S5
Assessment of the evaporation effect S5
Noise determination S6
Differences between simulated and artificial spectra S6
Ultrafiltration contaminants S7
Figures of merit of the PLSR of simulated and artificial datasets S7
Regression vector illustrating a minor overfitting S8
Correlation matrix for the in silico calibration S8

Mathematical Definitions

The Root Mean Square Error (RMSE) was defined as in Eq.S1

$$\mathrm{RMSE} = \sqrt{\frac{1}{N}\sum_{i=1}^{N}\left(y_i - \hat{y}_i\right)^2} \tag{S1}$$

where y_i is the reference albumin/creatinine value for sample i, $\hat{y}_i$ is the prediction obtained, and N is the total number of samples. The same formula applies for RMSE of cross-validation (RMSECV), where $\hat{y}_i$ is the prediction obtained when sample i belongs to the cross-validation fold, and.the RMSE of prediction (RMSEP), where $\hat{y}_i$ is the prediction obtained for samples in the validation set.

Variable Importance in Projection (VIP) scores[1] were defined as in Eq.S2

$$\mathrm{VIP_j} = \sqrt{p \cdot \frac{\sum_{k=1}^{h} \mathrm{SS}\left(b_k t_k\right)\left(\frac{w_{jk}}{||w_k||}\right)^2}{\sum_{k=1}^{h} \mathrm{SS}\left(b_k t_k\right)}} \tag{S2}$$

where p is the number of variables in the spectra, h is the number of LVs, w_{jk} is the weight of the variable j in the LV k, t_k are the X scores of the LV k and b_k is the regression coefficient for t_k.

Spectral shifts at high concentrations

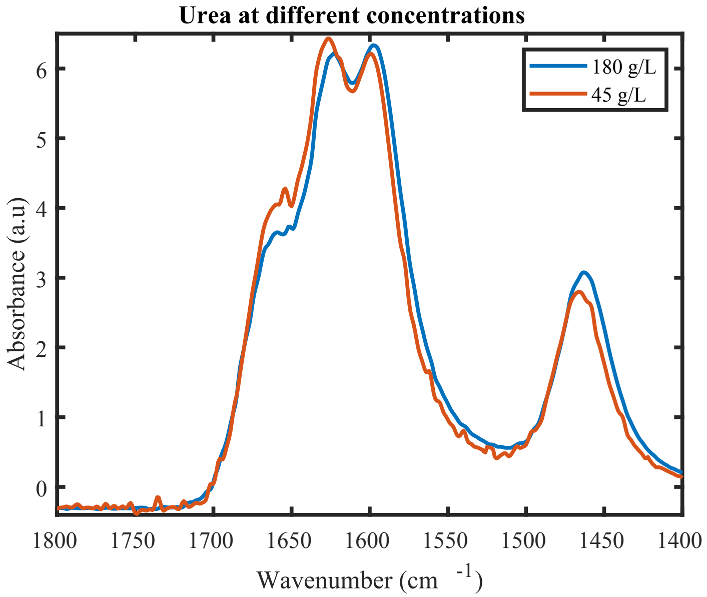

Figure S1: Urea spectra in aqueous solution at 180 g/L (blue) and 45 g/L (red) after water subtraction. Note that there is a slight shift in bands positions towards lower wavenumbers.

Individual spectra of the urine components

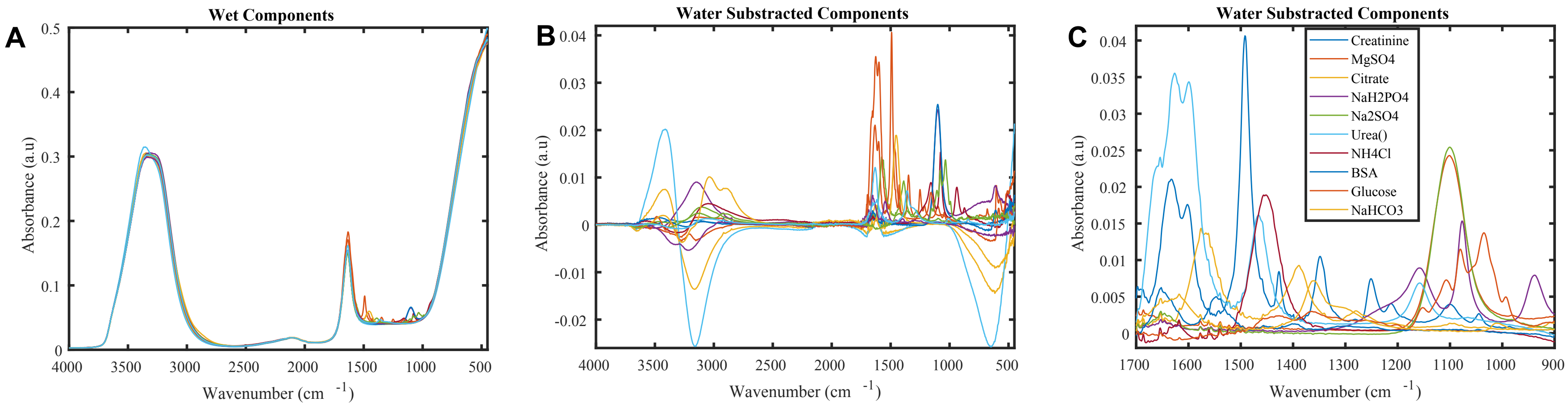

Figure S2: Water solutions of the individual urine components used for the generation of the *in silico* spectra before (A) and after water subtraction (B, C).

Assessment of the evaporation effect

The potential effect of evaporation during spectral acquisition was evaluated using the Spectrum Two instrument by measuring five replicates of 10 µL of a urea standard with 10 scans and five replicates of 10 µL with 50 scans. After correction for water contributions, an average increase of 3.2% in absorbance at the maximum signal intensity was observed. The difference in absorbance was reduced to 0.9% when the absorbance of 10 µL with 50 scans was compared to the absorbance of 3 µL with 10 scans (corresponding to the experimental conditions under which the standards and the urine samples, respectively, were measured with the Spectrum 2. This indicates that evaporation effects under the actual measurement conditions are minimal and are unlikely to significantly influence the analytical results.

Noise determination

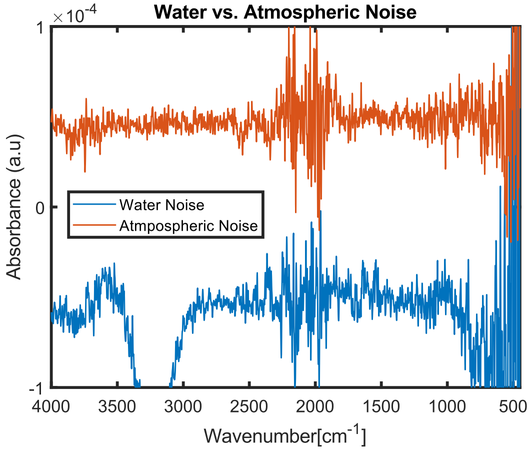

Figure S3. Mean (n=25) of the noise obtained when measuring the empty crystal (red) or a water sample (blue) with 10 scans. Spectra are offset for better visualization.

Differences between simulated and artificial spectra

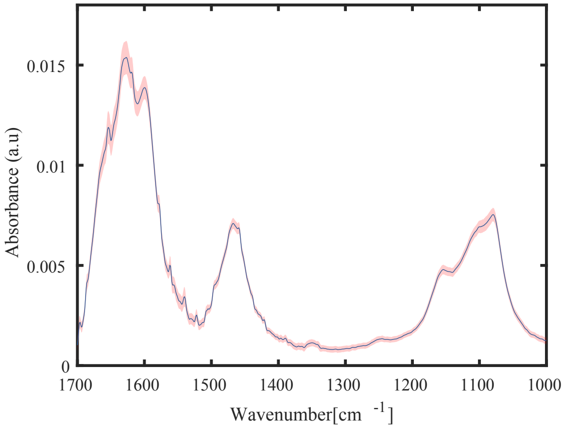

Figure S4. Mean artificial urine spectra, with shaded areas representing the root mean squared error (RMSE) of the residuals obtained from the linear fitting between simulated and artificial spectra.

Ultrafiltration contaminants

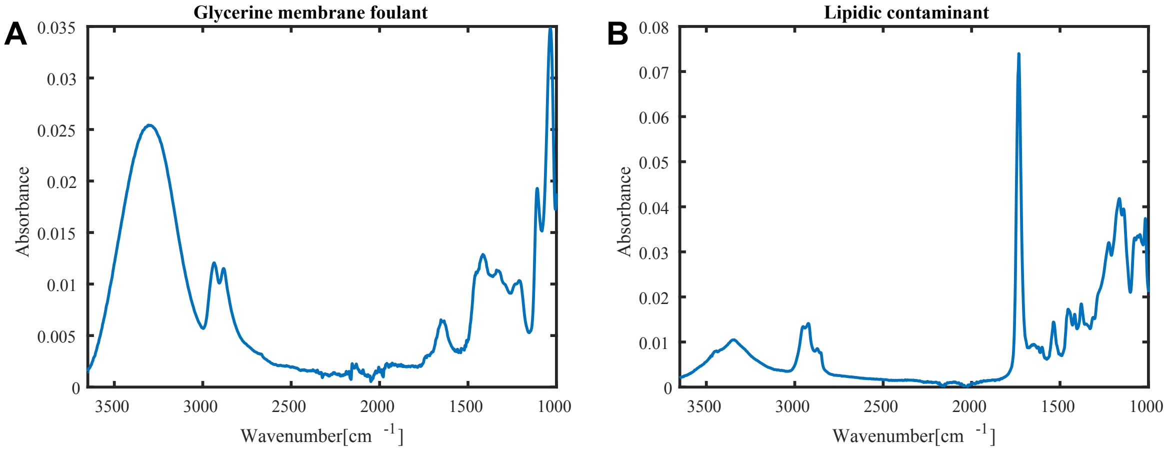

Figure S5: Dry Film spectra of the glycerine foulant from the 10kDa PES Vivaspin membrane obtained from the retained fraction of the pre-rinsing (A). Dry Film spectra of the isolated contaminant obtained after the ultrafiltration of one urine sample (B).

Figures of merit of the PLSR of simulated and artificial datasets

Table S1. RMSECV (mg/L), R^2 and LVs for albumin and creatinine in simulated and artificial urine at different experimental conditions

	Urine	1x1Sc			1x10Sc			25x2Sc			25x10Sc		
		RMSECV	R^2	LVs	RMSECV	R^2	LVs	RMSECV	R^2	LVs	RMSECV	R^2	LVs
Albumin	Simulated	103.3	0.221	1	103.3	0.242	1	29.8	0.911	4	29.5	0.913	4
	Artificial	101.7	0.012	2	101.9	0.007	2	34.5	0.881	4	33.6	0.887	4
Creatinine	Simulated	63.4	0.992	6	49.2	0.995	6	62.0	0.992	6	49.7	0.995	7
	Artificial	159.0	0.948	5	98.8	0.980	6	111.6	0.974	6	89.5	0.983	6

Regression vector illustrating a minor overfitting

Figure S6. Regression vectors of the PLS model 1x1Sc for creatinine calibrated using artificial (A) and simulated datasets (B).

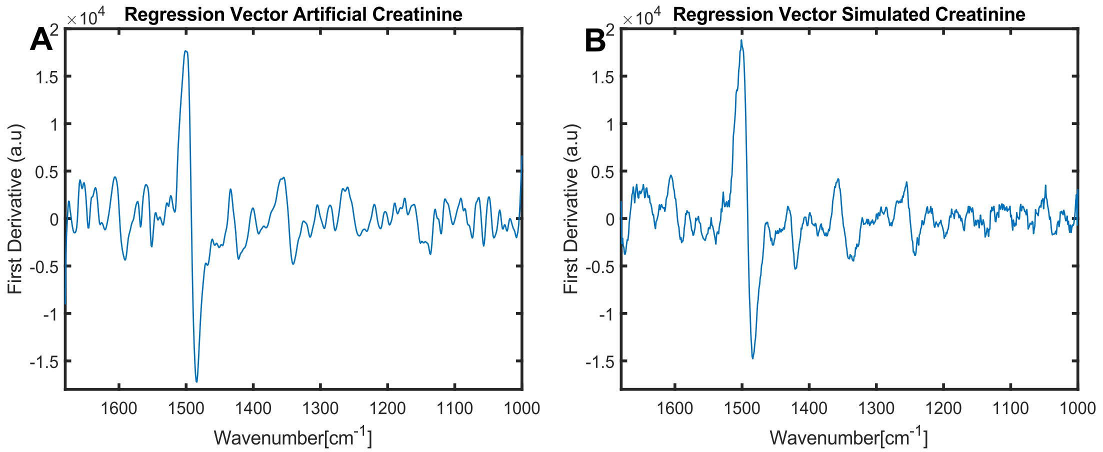

Correlation matrix for the in silico calibration

Table S2. Correlation matrix[2] for some components of urine used for the *in silico* calibration

	**Urea**	**Creatinine**	**Sulfate**	**Phosphate**
**Urea**	1.000	0.560	0.952	0.846
**Creatinine**	0.560	1.000	0.480	0.622
**Sulfate**	0.952	0.480	1.000	0.846
**Phosphate**	0.846	0.622	0.846	1.000

S References

(1) Chong, I.-G.; Jun, C.-H. Performance of Some Variable Selection Methods When Multicollinearity Is Present. *Chemometrics and Intelligent Laboratory Systems* **2005**, *78* (1), 103–112. https://doi.org/10.1016/j.chemolab.2004.12.011.

(2) Heise, H. M.; Voigt, G.; Lampen, P.; Küpper, L.; Rudloff, S.; Werner, G. Multivariate Calibration for the Determination of Analytes in Urine Using Mid-Infrared Attenuated Total Reflection Spectroscopy. *Applied Spectroscopy* **2001**, *55* (4), 434–443. https://doi.org/10.1366/0003702011951948.
